# Association of Burnout With Missed Nursing Care Among Neuro‐Oncology Nurses: The Mediating Role of Professional Identity and the Moderating Role of Psychological Capital

**DOI:** 10.1002/nop2.70527

**Published:** 2026-04-10

**Authors:** Li Ying, Duan Yuyu, Luo Huidan, Zhang Ping, Su Yangmei, Zhang Xiaowei, Zhou Zhihuan

**Affiliations:** ^1^ State Key Laboratory of Oncology in South China Guangzhou People's Republic of China; ^2^ Guangdong Provincial Clinical Research Center for Cancer Guangzhou People's Republic of China; ^3^ Department of Neurosurgery and Neuro Oncology Sun Yat‐sen University Cancer Center Guangzhou Guangdong People's Republic of China; ^4^ School of Nursing Guangzhou Medical University Guangzhou City Guangdong Province People's Republic of China

**Keywords:** burnout, missed nursing care, neuro‐oncology nurses, professional identity, psychological capital

## Abstract

**Aim:**

The aims of this study were to examine whether professional identity mediates the association between burnout and missed nursing care (MNC), and to test whether psychological capital moderates this mediation model.

**Design:**

A cross‐sectional study.

**Methods:**

The Oncology Missed Nursing Care Self‐rating Scale (OMNCS), Maslach Burnout Inventory (MBI), Professional Identification Scale (PIS) and Psychological Capital Scale (PCS) were used to assess 446 neuro‐oncology nurses from 10 Grade A oncology hospitals in six Chinese provinces between April and June 2024. Mediation and moderated mediation models were constructed using structural equation modelling.

**Results:**

The incidence of MNC among 446 neuro‐oncology nurses was 36.4%. The mean score on the OMNCS was 104.95 ± 26.70 (note: the OMNCS is reverse‐scored, with higher scores indicating fewer missed care occurrences). MBI showed a negative association with MNC (total effect: β = −0.605, *p* < 0.001), with a significant direct effect (β = −0.256, *p* < 0.001). PIS partially mediated this relationship (indirect effect: β = −0.349, *p* < 0.001), accounting for 57.7% of the total effect, through the paths ‘burnout → professional identity’ (β = −0.646, *p* < 0.001) and ‘professional identity → MNC’ (β = 0.540, *p* < 0.001). PCS moderated all three paths (β = −0.015, *p* < 0.001; β = −0.018, *p* < 0.05; β = 0.071, *p* < 0.05). As psychological capital increased from low to high, the indirect effect diminished in magnitude from −0.23 to −0.12. The moderated mediation model demonstrated good fit (GFI = 0.98, CFI = 0.97, RMSEA = 0.06, *χ* ^2^/df = 4.8).

**Conclusion:**

The direct correlation between burnout and heightened risk of MNC was evident, with burnout also exhibiting an indirect connection to MNC through the enhancement of professional identity in neuro‐oncology nurses. Psychological capital was identified as a potential moderator in offsetting the mediating impact of professional identity and in regulating the association between burnout and MNC. Future intervention strategies aimed at mitigating MNC among neuro‐oncology nurses experiencing burnout may benefit from enhancing professional identity and psychological capital. Such approaches could potentially improve care quality and patient outcomes.

**Patient or Public Contribution:**

No patient or public involvement occurred in the design, conduct, or reporting of this study. The study participants were neuro‐oncology nurses, and the research focused on their experiences and perceptions.

## 1 Introduction

Nursing is an important component of healthcare, and its quality directly influences patient safety (Lnghini et al. 2021); for example, nursing behaviour is associated with the incidence of patient mortality, falls, pressure ulcers and infections (Cho et al. 2015; Nelson and Flynn 2015; Gustafsson et al. 2020; Kalánková et al. 2020; Recio‐Saucedo et al. 2018). Missed nursing care (MNC) refers to the omission or delay in implementing nursing measures (Kalisch et al. 2009). Previous reports have indicated that 55%–98% of nurses have missed or not completed at least one nursing measure at work (Cho et al. 2016; Jones et al. 2015; Haftu et al. 2019), while the miss rate for basic nursing and care planning exceeds 70% (Kalisch and Xie 2014).

Patients with brain tumours are susceptible to brain hernia, epilepsy and neurological impairment during hospitalization. When necessary nursing measures are not completed due to MNC, the quality of care provided by neuro‐oncology nurses decreases, which has been associated with poorer treatment outcomes (Recio‐Saucedo et al. 2018; Min et al. 2020). Furthermore, the complexity of neuro‐oncology nursing means that directly addressing job burnout may not be the most effective strategy for preventing MNC. Identifying moderators to support multifaceted interventions could be more advantageous. Thus, identifying potential mediating pathways and modifiable factors may provide valuable insights for developing strategies to reduce MNC.

MNC is the result of multiple complex factors and is proposed to be linked to negative emotions associated with burnout (Kalisch et al. 2009). Nurses experiencing burnout tend to report a higher incidence of MNC (Lake, Staiger, et al. 2020; Tubbs‐Cooley et al. 2019). A cross‐sectional study has reported that American registered nurses with burnout were five times more likely to leave necessary care undone than those without burnout (Molina‐Praena et al. 2018). Additionally, nurses in good work environments were ten times less likely to report job dissatisfaction and eight times less likely to experience burnout compared to those in poor environments (Singh 2019). Moreover, MNC due to insufficient time, limited resources, or poor work environments is common in nursing practice and is associated with burnout and job dissatisfaction (Molina‐Praena et al. 2018; Singh 2019).

According to the missed nursing care model (Kalisch et al. 2009), nurses' internal cognitive states can affect MNC, and professional identity and psychological capital are considered to play important roles in these cognitive processes (White et al. 2019). Previous cross‐sectional (White et al. 2020) and qualitative studies (Bakker 2018) have reported that nurses with high professional identity have significantly lower levels of MNC. Some studies have suggested that high professional identity may improve job satisfaction and nursing quality, thereby reducing MNC (Xu et al. 2022; Keshmiri et al. 2020). In contrast, low professional identity may increase turnover intention and MNC. Furthermore, reducing burnout can lower the risk of MNC, and professional identity has been identified as the most critical mediator in this process (Bakker 2018).

Professional identity may be enhanced through internal factors, such as psychological capital (Pizziconi et al. 2021). Studies have indicated that individuals with higher psychological capital tend to adapt better to burnout and may experience fewer of its adverse effects (Chen et al. 2021; Ackerhans et al. 2024). Additionally, according to stress process theory (Ren et al. 2021), the demanding nature of nursing work precipitates higher burnout levels, which may trigger stress responses such as MNC. Therefore, maintaining high psychological capital is crucial for neuro‐oncology nurses to effectively manage stress. However, existing literature inadequately validates the mediating role of professional identity and the moderating effect of psychological capital in the relationship between burnout and MNC.

Neuro‐oncology nurses consistently care for patients with poor prognoses and high symptom burdens, working in an environment marked by significant emotional challenges and uncertainty. These persistent stressors are associated with emotional exhaustion, which may subsequently be related to the comprehensiveness and quality of nursing care (Völz et al. 2024). Studies have shown (Völz et al. 2024; Galanis, Moisoglou, et al. 2025; Ying et al. 2025) that when addressing patients' psychological distress, neuro‐oncology nurses often develop occupational burnout due to factors such as time constraints and heavy emotional burdens, which can further contribute to MNC.

Previous studies on MNC have focused on nurses in ICUs, paediatrics and gynaecology, while research on neuro‐oncology nurses remains scarce (Liu et al. 2023; Creswell et al. 2015). MNC among neuro‐oncology nurses has received limited attention, and the roles of professional identity and psychological capital in the relationship between burnout and MNC are poorly understood. Therefore, understanding and addressing MNC is crucial for improving care quality and ensuring patient safety. In this study, we explored the moderated mediating effects of professional identity and psychological capital on the relationship between burnout and MNC among neuro‐oncology nurses.

## 2 Materials and Methods

### 2.1 Study Participants and Procedures

A cross‐sectional survey of neuro‐oncology nurses from 10 Grade A oncology hospitals in six Chinese provinces (Guangxi, Shanghai, Henan, Tianjin, Guangdong and Chongqing) was conducted from April to June 2024. All neuro‐oncology nurses were selected to participate in a self‐administered questionnaire survey using convenience sampling. Nurse participant inclusion criteria were: (1) a qualified clinical nurse practitioner working in a neuro‐oncology department (nursing managers and unit leaders were excluded); (2) had been working for at least 1 year; (3) demonstrated an understanding of the research procedures and could read and communicate in Chinese. The exclusion criteria were: (1) nurses who were on leave; (2) nurses who did not participate in clinical treatment.

We forwarded the survey links to the neuro‐oncology nurses and requested their voluntary participation. Prior to data collection, all potential participants were provided with detailed information about the study's purpose, procedures, potential benefits and risks. They were assured of the voluntary nature of participation and the confidentiality of their responses. Written informed consent was obtained from all participants before they accessed the online survey. The study strictly followed the principles of informed consent, and all procedures were conducted in accordance with ethical standards for human subjects research.

### 2.2 Measurements

#### 2.2.1 General Information Questionnaire

A general information questionnaire was used to collect details of the characteristics of participants, including: age, sex, education, marital status, working hours per shift, working years, weekly overtime, professional title, night shifts per month, shift situation and job satisfaction.

#### 2.2.2 Oncology Missed Nursing Care Self‐Rating Scale

The Oncology Missed Nursing Care Self‐rating Scale (OMNCS) (Liu et al. 2019) is widely used in China and has high levels of reliability and validity and was used to evaluate the impact of MNC among oncology nurses. The scale is a 33‐item scale covering four domains: nursing assessment, care planning, primary care and nursing intervention. Each item was rated on a 5‐point scale from 1 (always have been) to 5 (never before). Cronbach's α for the scale in this study was 0.95, and the test–retest reliability was 0.90. Total scores ranged from 30 to 180. It is important to note that the OMNCS is reverse‐scored: higher scores indicate a lower frequency of missed nursing care, while lower scores indicate more frequent missed nursing care. Therefore, a negative association with OMNCS means that higher burnout is related to more missed nursing care; conversely, a positive association with OMNCS means that higher professional identity is related to less missed nursing care. This scoring direction should be kept in mind when interpreting all subsequent results.

#### 2.2.3 Maslach Burnout Inventory

Burnout was measured using the Chinese Version of the Maslach Burnout Inventory (MBI) (Feng et al. 2004), which is a 22‐item scale covering three domains: emotional exhaustion, depersonalization and personal achievement. The items are presented in a 7‐point Likert format, ranging from ‘never have’ to ‘every day’, with total scores ranging from 22 to 154. Higher scores for the emotional exhaustion and depersonalization domains and lower scores for the personal achievement domain indicated higher levels of burnout. Cronbach's α for the scale in this study was 0.823, and test–retest reliability was 0.861.

#### 2.2.4 Professional Identification Scale

The Chinese Version of the Professional Identification Scale (PIS) consists of 30 items covering five domains (Liu et al. 2011): occupational cognitive evaluation, occupational social support, occupational social skills, occupational frustration coping, and occupational self‐reflection. Each item is rated on a 5‐point scale from 1 (strongly disagree) to 5 (strongly agree); total scores range from 30 to 150. Higher total scores indicate a greater impact of professional identity. Cronbach's α for the scale in this study was 0.94, and test–retest reliability was 0.90.

#### 2.2.5 Psychological Capital Scale

The Chinese Version of the Psychological Capital Scale (PCS) was used to evaluate the psychological impact of psychological capital on nurses during the past week (Yuan et al. 2023). The PCS is a 30‐item measure covering six domains: hope, assist in communication, emotional quotient, sense of responsibility, toughness and self‐confidence. Items are presented in a 6‐point Likert format, ranging from ‘strongly disagree’ to ‘strongly agree’. Total scores ranged from 30 to 180, with higher total scores indicating a greater impact of psychological capital. A total score below 90 points indicates low psychological capital, a score between 90 and 120 points indicates medium psychological capital, and a score above 120 points indicates high psychological capital. Cronbach's α for the scale in this study was 0.91, and test–retest reliability was 0.94.

### 2.3 Sample Size

According to Kendall's empirical rule (Meyer and Morin 2016), this study included 29 independent variables, comprising 11 variables from general information, as well as 4 dimensions from the oncology missed nursing care self‐rating scale, 3 dimensions related to the maslach burnout inventory, 5 dimensions related to the professional identification scale and 6 dimensions from the psychological capital scale. To ensure robust statistical validity, the sample size was calculated to be 10–15 times the number of variables, while also accounting for a potential 10% sample loss; the required sample size for the study should be between 323 and 484. Our study included 446 participants, meeting the specified sample size requirements.

### 2.4 Data Analysis

Descriptive analysis was employed to depict the sociodemographic attributes of participants and key study variables, while the comparison of MNC distribution was conducted through *t*‐tests and chi‐square tests using SPSS 27.0 (IBM; Armonk, New York, NY). The interrelations among the participants' MNC, burnout, professional identity and psychological capital were scrutinized through the bivariate Pearson correlation coefficient. Subsequently, a mediation model was formulated and the bootstrap method was employed to assess the mediating effect. *χ* ^2^/df, goodness‐of‐fit index (GFI), comparative fit index (CFI), Tucker‐Lewis index (TLI), the normed fit index (NFI), and root mean square error of approximation (RMSEA) were used for model fit evaluation. Mplus 8.3 (Muthén; Los Angeles, CA) was employed to examine the moderating influence of psychological capital within the mediation model. In this study, the moderating effects of psychological capital on both mediating and direct effects were concurrently assessed. Subsequently, leveraging the Johnson‐Neyman method introduced by Hayes and Rockwood (2017), conditional effects and confidence bands that vary with the moderator were graphically depicted.

## 3 Results

### 3.1 Neuro‐Oncology Nurses Characteristics

Of the 446 neuro‐oncology nurses who participated in this study, the largest proportion, 271 (60.8%), were aged 18–35 years. A total of 415 (93%) were female, while 31 (7%) were male. Additionally, 248 (55.6%) were married, and 354 (79.4%) held a college degree or higher. Among the participants, 208 (46.6%) were nurse supervisors, 154 (34.5%) had 11–20 years of experience, 268 (60.1%) worked 9–15 h per shift, and 275 (61.7%) reported being generally satisfied with their jobs (Table 1).

**TABLE 1:** Participant neuro‐oncology nurse characteristics (*N* = 446).

Variable	Group	*n* (%)	OMNCS score, mean ± SD	*F/t*	*p*
Sex	Male	31 (7.0)	92.84 ± 25.66	0.900^a^	−2.636
	Female	415 (93.0)	105.86 ± 26.58		
Age (years)	18–25	85 (19.1)	88.40 ± 27.90	31.284^b^	< 0.001
	26–35	184 (41.3)	99.92 ± 19.61		
	36–45	130 (29.1)	113.78 ± 23.82		
	46–55	34 (7.6)	125.91 ± 30.45		
	> 55	13 (2.9)	141.23 ± 25.03		
Marital status	Unmarried	195 (43.7)	105.04 ± 26.68	2.210^b^	0.111
	Married	248 (55.6)	104.50 ± 26.69		
	Divorced	3 (0.7)	137.00 ± 4.00		
Education	Technical secondary school	92 (20.6)	104.74 ± 19.24	6.635^b^	0.001
	College degree	339 (76.0)	103.93 ± 28.22		
	Graduate student or above	15 (3.4)	129.27 ± 18.94		
Professional title	Nurse	78 (17.5)	91.19 ± 29.16	16.270^b^	< 0.001
	Nurse practitioner	139 (31.2)	100.40 ± 21.39		
	Supervisor nurse	208 (46.6)	111.67 ± 25.55		
	Deputy chief nurse or above	21 (4.7)	119.62 ± 33.08		
Working hours per shift (h)	≤ 8	178 (39.9)	115.69 ± 24.90	62.580^b^	< 0.001
	9–12	237 (53.1)	101.99 ± 22.44		
	13–15	31 (7.0)	65.90 ± 25.00		
Number of night shifts per month	≤ 2	140 (31.4)	107.16 ± 28.76	13.312^b^	< 0.001
	3–6	278 (62.3)	106.30 ± 24.86		
	7–10	28 (6.3)	80.46 ± 21.96		
Weekly overtime	Seldom	71 (15.9)	110.55 ± 23.02	1.599^b^	0.189
	Now and then	239 (53.6)	104.96 ± 28.26		
	Frequently	128 (28.7)	102.03 ± 25.10		
	Always	8 (1.8)	101.63 ± 30.82		
Shift situation	Regular day shift	75 (16.8)	100.28 ± 27.80	0.791^a^	−1.664
	Take turns	371 (83.2)	105.89 ± 26.41		
Working years	1–5	120 (26.9)	105.39 ± 28.53	0.218^b^	0.884
	6–10	126 (28.3)	103.90 ± 25.58		
	11–20	154 (34.5)	104.70 ± 26.91		
	> 20	46 (10.3)	107.50 ± 24.60		
Job satisfaction	Dissatisfied	101 (22.6)	93.16 ± 23.67	15.076^b^	< 0.001
	Normal	275 (61.7)	107.17 ± 24.30		
	Satisfied	70 (15.7)	113.24 ± 33.88		
Burnout			128.02 ± 40.24	66.533	< 0.001
Professional identity			79.36 ± 21.56	1.535	0.008
Psychological capital			117.37 ± 43.90	6.129	< 0.001

Abbreviations: *F*, variance test; OMNCS, Oncology Missed Nursing Care Self‐rating Scale; *p*, *p* value; SD, standard deviation; *t*, *t*‐test statistic value.

^a^ *t*‐test.

^b^ *F*.

### 3.2 Scores for Each Scale

Mean ± standard deviation (SD) score for the OMNCS measuring MNC among neuro‐oncology nurses was 104.95 ± 26.70, while corresponding scores for the nursing assessment, care planning, primary care, and nursing intervention domains were 17.21 ± 5.23, 17.45 ± 4.75, 25.60 ± 8.17 and 44.70 ± 15.47, respectively. Mean ± SD burnout score, assessed using the MBI, was 128.02 ± 40.24, with scores for the emotional exhaustion, depersonalization, and personal achievement domains of 34.26 ± 13.73, 17.66 ± 7.66 and 24.19 ± 8.40, respectively. Mean ± SD professional identity (PIS) and psychological capital (PCS) scores were 79.36 ± 21.56 and 117.37 ± 43.90, respectively. Differences in MNC were detected according to participant age, education level, professional title, working hours per shift, night shifts per month and job satisfaction (Table 1). It should be noted that the OMNCS is reverse‐scored; therefore, higher scores indicate lower frequency of MNC. This scoring direction should be considered when interpreting the correlations and regression coefficients reported below.

### 3.3 Correlations of Variables

The OMNCS is reverse‐scored, meaning that higher scores indicate a lower frequency of MNC. Therefore, when interpreting the correlations below, a negative correlation with OMNCS indicates an association with more MNC, while a positive correlation indicates an association with less MNC. Partial correlations between total scores of core variables, as well as their dimensional correlations (adjusted for confounding variables), are presented in Table 2. Regarding total scale correlations: MNC (OMNCS total score) was negatively correlated with burnout (MBI total score, *r* = −0.41, *p* < 0.001) and positively correlated with professional identity (PIS total score, *r* = 0.24, *p* < 0.001) and psychological capital (PCS total score, *r* = 0.36, *p* < 0.001). Burnout (MBI total score) was negatively correlated with both professional identity (*r* = −0.22, *p* < 0.001) and psychological capital (*r* = −0.43, *p* < 0.001), while professional identity and psychological capital showed a weak positive correlation (*r* = 0.10, *p* < 0.05). Regarding dimensional correlations: All four dimensions of MNC were negatively correlated with emotional exhaustion (*r* = −0.46 to −0.56, *p* < 0.001) and depersonalization (*r* = −0.42 to −0.69, *p* < 0.001) but positively correlated with personal achievement (*r* = 0.35–0.51, *p* < 0.001). Professional identity and psychological capital were positively correlated with all MNC dimensions (*r* = 0.17–0.43, *p* < 0.05 or *p* < 0.001) and personal achievement (*r* = 0.13–0.33, *p* < 0.05 or *p* < 0.001), while negatively correlated with emotional exhaustion (*r* = −0.16 to −0.38, *p* < 0.05 or *p* < 0.001) and depersonalization (*r* = −0.24 to −0.45, *p* < 0.001).

**TABLE 2:** Partial correlations among missed nursing care, burnout, professional identity and psychological capital.

Variable	1	2	3	4	5	6	7	8	9	10	11
1. OMNCS total	1										
2. Nursing assessment	0.82**	1									
3. Care planning	0.80**	0.44**	1								
4. Primary care	0.85**	0.41**	0.50**	1							
5. Nursing intervention	0.88**	0.21**	0.31**	0.72**	1						
6. MBI total	−0.41**	−0.39**	−0.38**	−0.52**	−0.50**	1					
7. Emotional exhaustion	−0.39**	−0.46**	−0.45**	−0.56**	−0.53**	0.89**	1				
8. Depersonalization	−0.40**	−0.42**	−0.44**	−0.66**	−0.69**	0.87**	0.90**	1			
9. Personal achievement	0.38**	0.35**	0.42**	0.51**	0.44**	−0.32**	−0.25**	−0.30**	1		
10. PIS total	0.24**	0.17**	0.20**	0.22**	0.27**	−0.22**	−0.16**	−0.24**	0.13*	1	
11. PCS total	0.36**	0.23**	0.28**	0.39**	0.43**	−0.43**	−0.38**	−0.45**	0.33**	0.10*	1

*Note:* (1) Adjusted for age, education, professional title, working hours per shift, night shifts per month and job satisfaction; (2) Items 2, 3, 4 and 5 are the four dimensions of the OMNCS; Items 7, 8 and 9 are the three dimensions of the MBI; (3) *p < 0.05, **p < 0.001. (4) Higher OMNCS score indicates lower frequency of missed nursing care (MNC); higher MBI total score indicates higher burnout; higher PIS/PCS total scores indicate stronger professional identity/psychological capital. Please note the reverse scoring of OMNCS when interpreting correlation directions.

Abbreviations: MBI, Maslach Burnout Inventory; OMNCS, Oncology Missed Nursing Care Self‐rating Scale; PCS, Psychological Capital Scale; PIS, Professional Identification Scale.

### 3.4 Mediated Moderation Analysis

The mediation model exhibited a favourable fit to the data, as evidenced by the indicators GFI = 0.98, CFI = 0.97, TLI = 0.96, RMSEA = 0.06 and *χ* ^2^/df = 4.8. The mediation analysis revealed a statistically significant total effect of burnout on MNC (β = −0.605, *p* < 0.001). Moreover, notable effect coefficient values were observed for the direct pathways: burnout → professional identity (β = −0.646, *p* < 0.001) and professional identity → MNC (β = 0.540, *p* < 0.001), as well as for the indirect pathway: burnout → professional identity → MNC (β = −0.349, *p* < 0.001), indicating the mediating role of professional identity in the relationship between burnout and MNC. The direct impact coefficient of burnout on MNC also yielded significance (β = −0.256, *p* < 0.001), suggesting that professional identity served as a partial mediator in the association between burnout and MNC, accounting for 57.7% of the total effect (Table 3).

**TABLE 3:** Standardized direct effects, indirect effect and total effect of the mediation model.

	Model pathway	Effect	LLCI	ULCI
Direct effect	Burnout → Professional identity	−0.646**	−0.776**	−0.526**
	Professional identity → Missed nursing care	0.540**	0.420**	0.631**
	Burnout → Missed nursing care	−0.256**	−0.386**	−0.161**
Indirect effect	Burnout → Professional identity → Missed nursing care	−0.349**	−0.476**	−0.222**
Total effect	Burnout → Missed nursing care**	−0.605**	−0.729	−0.481**
*R* ^2^ = 0.381 *p <* 0.001				

*Note:* (1) Adjusted for age, education, professional title, working hours per shift, night shifts per month and job satisfaction; (2) **p < 0.001.

Abbreviations: LLCI, lower level of the confidence interval; ULCI, upper level of the confidence interval.

**FIGURE 1:**
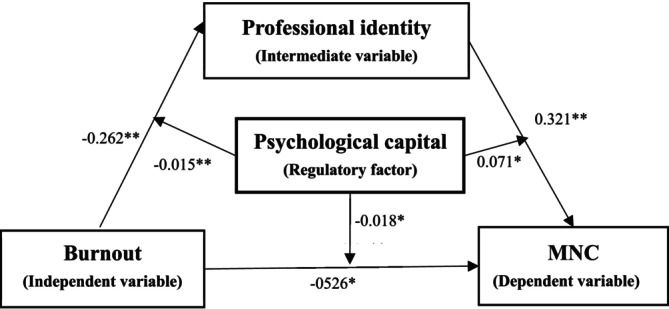
Moderated mediation model illustrating the associations among burnout, professional identity, psychological capital and MNC among neuro‐oncology nurses. (1) Adjusted for age, education, professional title, working hours per shift, night shifts per month and job satisfaction. (2) MNC, missed nursing care. (3) The OMNCS is reverse‐scored; higher scores indicate fewer missed nursing care incidents. Therefore, negative coefficients indicate associations with more missed care, while positive coefficients indicate associations with less missed care. (4) The indirect effect of burnout on MNC through professional identity was β = −0.349 (95% CI [−0.476, −0.222], *p* < 0.001), accounting for 57.7% of the total effect. (5) *p < 0.05, **p < 0.001.

The outcomes derived from the moderated mediation model analysis are encapsulated in Figure 1. Psychological capital demonstrated substantial influences on three regression pathways, specifically: burnout→ professional identity (β = −0.015, *p* < 0.001); burnout → MNC (β = −0.018, *p* < 0.05); and professional identity → MNC (β = 0.071, *p* < 0.05). These results suggest that psychological capital may buffer the negative association between burnout and professional identity, weaken the direct relationship between burnout and MNC, and attenuate the positive association between professional identity and MNC.

The additional findings derived from the moderated mediation model analysis are presented in Table 4. This analysis delved into the indirect and direct impacts of burnout on MNC at low (mean‐SD), moderate (mean), and high (mean + SD) levels of psychological capital. Notably, the indirect effects of ‘burnout → professional identity → MNC’ were significant across all three psychological capital levels. Moreover, the magnitude of the indirect effect coefficients reflecting the influence of burnout on MNC exhibited a decline from −0.23 to −0.12 (i.e., becoming less negative) as the level of psychological capital increased from low to high, suggesting that higher psychological capital may attenuate the mediating role of professional identity (Table 4).

**TABLE 4:** Conditional indirect effects of burnout on missed nursing care at different levels of psychological capital.

Psychological capital level	Effect	BootSE	*p*	BootLLCI	BootULCI
Low	−0.23	0.03	< 0.05	−0.21	−0.39
Moderate	−0.17	0.01	< 0.05	−0.15	−0.24
High	−0.12	0.01	< 0.05	−0.09	−0.15

*Note:* Adjusted for age, education, professional title, working hours per shift, night shifts per month and job satisfaction.

Abbreviations: Boot, bootstrap; LLCI, lower levels of the confidence interval; *p*, *p* value; SE, standard error; ULCI, upper level of the confidence interval.

**FIGURE 2:**
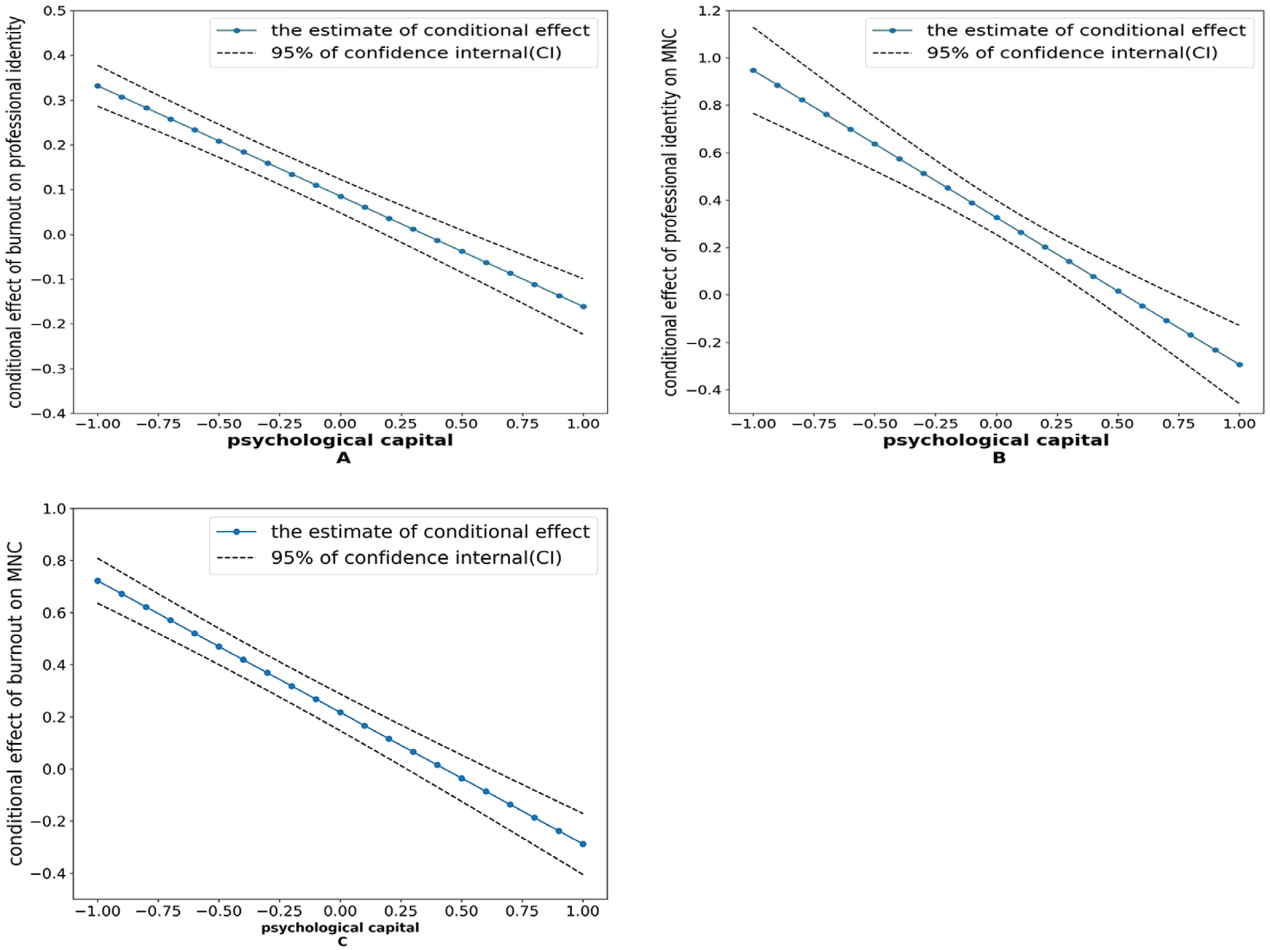
Conditional effects of the moderated mediation model, showing how the associations among burnout, professional identity and MNC vary across levels of psychological capital. (1) Adjusted for age, education, professional title, working hours per shift, night shifts per month and job satisfaction; (2) Psychological capital was standardized; (3) MNC, missed nursing care; (4) (A) The conditional effects of burnout on professional identity diminish with rising psychological capital, with none of the 95% CI values encompassing zero; (5) (B) The conditional effects of professional identity on MNC diminish with rising psychological capital, with none of the 95% CI values encompassing zero; (6) (C) The conditional effects of burnout on MNC diminish with rising psychological capital, with none of the 95% CI values encompassing zero.

The Figure 2 illustrates the conditional effects of ‘burnout → professional identity’, ‘burnout → MNC’ and ‘professional identity → MNC’. These three conditional effects exhibited a decline as psychological capital levels increased, with all 95% confidence interval (CI) values excluding zero. These outcomes suggest that psychological capital, regardless of its magnitude, has the capacity to moderate the connections observed in ‘burnout → professional identity’, ‘burnout → MNC’ and ‘professional identity → MNC’.

## 4 Discussion

In this study, we examined the relationships among burnout, professional identity, psychological capital and MNC in neuro‐oncology nurses. The findings revealed a negative correlation between burnout and MNC in this professional group, with professional identity serving as a mediator in the link between burnout and MNC. Furthermore, psychological capital was found to moderate these relationships. Particularly noteworthy was the observation that higher levels of psychological capital were associated with a weaker direct relationship between burnout and MNC, as well as a reduced mediating role of professional identity.

The incidence of MNC among neuro‐oncology nurses was found to be 36.4%, which are identical results to those reported previously (Cho et al. 2016; Jones et al. 2015; Haftu et al. 2019), suggesting that MNC warrants serious consideration in this context. Brain tumours have a high degree of malignancy and patients with these cancers undergo rapid changes in their health status; the necessary nursing work involved is complex. Further, the proportion of nurses to patients is insufficient, the workload is large, and medical communication and cooperation may not occur in a timely manner. The results of previous studies have provided evidence that oncology nurses experience significant stress during their clinical work, including with respect to workload and time allocation, the working environment and resources, nursing profession and career issues, and patient care (Boyle 2015; Soheili et al. 2021; Zaghini et al. 2020). Further, MNC in neuro‐oncology departments may cause more harm than in other oncology departments (Chaboyer et al. 2021), affecting patient outcomes.

In this study we explored that burnout is negatively correlated with MNC among neuro‐oncology nurses, similar to the findings of previous research (Lake, Staiger, et al. 2020; Tubbs‐Cooley et al. 2019; Molina‐Praena et al. 2018). MNC due to burnout and job dissatisfaction is common in nursing work. Therefore, interventions aimed at reducing burnout may be beneficial in lowering the risk of MNC.

We also discovered that professional identity played a partial mediating role in the connection between burnout and MNC among neuro‐oncology nurses. According to the missed nursing care model (Kalisch et al. 2009), when personal characteristics of nurses, such as professional identity and other internal motivations are higher, neuro‐oncology nurses may actively work to ensure care quality and patient safety and reduce MNC. Our results were consistent with this theory and the results of previous studies, indicating that professional identity is an important factor in inducing MNC among neuro‐oncology nurses (White et al. 2020; Bakker 2018; Xu et al. 2022; Keshmiri et al. 2020). In addition, we found that neuro‐oncology nurses who had experienced burnout were more likely to have low levels of professional identity, where professional identity acted as a mediating factor in the relationship between burnout and MNC. These findings suggest that professional identity may represent an internal mechanism linking burnout to MNC.

Our findings suggested that psychological capital possesses the capability to moderate the mediating influence of professional identity between burnout and MNC, alongside the direct route from burnout to MNC among neuro‐oncology nurses. With the elevation in psychological capital levels, a gradual weakening was observed in the mediating impact of professional identity and the direct influence of burnout on MNC. These results are consistent with the missed nursing care model (Kalisch et al. 2009), suggesting that professional identity, in the context of burnout, may interact with low psychological capital to be associated with higher MNC.

According to stress process theory (Ren et al. 2021), individuals with elevated psychological capital are adept at managing burnout effectively. Previous studies have posited that heightened levels of psychological capital have the capacity to alleviate the detrimental consequences of burnout, thereby diminishing turnover intention and reducing job stress (Duan et al. 2017; Kim and Kweon 2020; Tang et al. 2023; Ren et al. 2021). Additionally, a cross‐sectional study (Okros and Vîrgă 2022) has indicated that individuals with elevated psychological capital exhibit enhanced abilities to navigate their work environment efficiently, rendering them less susceptible to negative responses to unfavourable circumstances.

Moreover, our study revealed that psychological capital played a moderating role in the correlation between professional identity and MNC. Neuro‐oncology nurses exhibiting elevated psychological capital are known to display optimistic attitudes towards self‐efficacy and professional identity. They maintain the belief in their capacity to navigate burnout and stress effectively, leveraging supportive resources to enhance the quality of care. As a result, these nurses were reported to be less prone to experiencing MNC (Ren et al. 2021; Okros and Vîrgă 2022; Kim and Lee 2020). In addition, our findings showed that psychological capital can moderate the relationship between burnout and MNC, consistent with previous investigations (Molina‐Praena et al. 2018; Singh 2019; Chen et al. 2024). Burnout of nurses can lead to decreased work efficiency, increased absenteeism, personnel turnover and MNC. Previous studies have found that the psychological capital of clinical nurses can lower the rate of burnout (Kim and Kweon 2020; Tang et al. 2023; Ren et al. 2021); however, these correlations did not prove causal relationships, and further research is warranted.

Neuro‐oncology nurses face multidimensional stressors stemming from caring for brain tumour patients, including managing complex neurological symptoms, responding to patients' high emotional needs, and the inner guilt associated with the inherent ‘chronic unpredictability’ of oncology care (Galanis, Moisoglou, et al. 2025; Galanis, Katsiroumpa, et al. 2025). This high‐pressure environment significantly depletes nurses' psychological capital and reduces their job satisfaction (Völz et al. 2024). Recent research (Li and Zhou 2024) has suggested that this may constitute a critical pathway associated with missed nursing care and burnout: when psychological resources were exhausted, nurses could reduce their work engagement due to emotional exhaustion or a ‘quiet quitting’ mentality, which may be associated with increased risk of care omissions and could contribute to a cycle of burnout.

Our exploration of the potential internal mechanism mediating the relationship between job burnout and MNC among neuro‐oncology nurses had important implications for future research and intervention. Regarding neuro‐oncology departments, nursing managers may consider appropriate allocation of human resources and equipment (Cho et al. 2015; Völz et al. 2024; Du et al. 2020), as well as provision of psychological support (Park et al. 2018). Support groups for neuro‐oncology nurses can provide opportunities for those who experience burnout to connect and talk about work stress and difficulties at work, as well as appropriate solutions, potentially mitigating the adverse effects of burnout (Schubert et al. 2021). Regarding hospitals, managers should provide more opportunities for nurses to participate in discussions about hospital safety and service quality (Park et al. 2018). Improved working environments may strengthen nurses' sense of professional mission, which could in turn enhance professional identity and be associated with lower MNC (Park et al. 2018; Lake, Riman, and Sloane 2020; Kim et al. 2018; Smith et al. 2018).

The current study encountered several limitations. Firstly, the recruitment of participants from ten specific cancer centres deviated from random sampling methods. To enhance research validity, subsequent studies are encouraged to employ stratified and random cluster sampling techniques. Secondly, our investigation solely explored MNC among neuro‐oncology nurses at a single time point, thereby failing to capture the dynamic fluctuations in MNC over time. Additionally, the causal interconnections among professional identity, psychological capital, burnout and MNC remain inadequately delineated. A longitudinal study is imperative to decipher the evolving trajectory of MNC within the domain of neuro‐oncology nursing. Lastly, pivotal variables that could potentially impact the studied relationships were omitted in the current study. These variables warrant further investigation in future research endeavours.

## 5 Conclusion

Burnout exhibits a negative correlation with MNC, wherein professional identity plays a partial mediating role in this relationship. Furthermore, higher levels of psychological capital were associated with a weaker mediating role of professional identity and a weaker direct association between burnout and MNC. Our study provides a foundation for understanding MNC among neuro‐oncology nurses and suggests that enhancing psychological capital and professional identity may be beneficial in addressing burnout and MNC in this population. However, given the cross‐sectional design, these findings should be confirmed through longitudinal or interventional studies.

## Author Contributions

Z.Z. and L.Y. contributed to the conception and design of the study. D.Y., L.H., Z.X., Z.P., S.Y. and L.Y. undertook material preparation, data collection and analysis. The initial manuscript draft was composed by L.Y., while Z.Z. provided feedback on prior versions. All authors reviewed and endorsed the final manuscript.

## Funding

This work was supported by the Guangdong Nurses Association Scientific Research Fund Project, China [grant number gdshsxh2024qn14] and the Nurturing funds for nursing young talents of Sun Yat‐sen University, China [grant number N2022Y05].

## Ethics Statement

This study was approved by the hospital ethics committee (Approval No. SL‐B2022‐416‐02). All procedures performed were in accordance with the ethical standards of the institutional research committee and with the 1964 Helsinki Declaration and its later amendments. Written informed consent was obtained from all individual participants included in the study.

## Consent

Written informed consent was obtained from all participants prior to their enrollment in the study. Participants were provided with detailed information about the study's purpose, procedures, potential risks and benefits, and they were assured of confidentiality and the right to withdraw at any time without consequence.

## Conflicts of Interest

The authors declare no conflicts of interest.

## Data Availability

The data that support the findings of this study are available on request from the corresponding author. The data are not publicly available due to privacy or ethical restrictions.
